# Tracking Performance in Endurance Racing Sports: Evaluation of the Accuracy Offered by Three Commercial GNSS Receivers Aimed at the Sports Market

**DOI:** 10.3389/fphys.2018.01425

**Published:** 2018-10-09

**Authors:** Øyvind Gløersen, Jan Kocbach, Matthias Gilgien

**Affiliations:** ^1^Condensed Matter Physics, Department of Physics, University of Oslo, Oslo, Norway; ^2^Department of Physical Performance, Norwegian School of Sport Sciences, Oslo, Norway; ^3^Centre for Elite Sports Research, Department of Neuromedicine and Movement Science, Faculty of Medicine and Health Sciences, Norwegian University of Science and Technology, Trondheim, Norway; ^4^Norwegian Ski Federation, Alpine Skiing, Oslo, Norway

**Keywords:** global navigation satellite systems, GPS, speed, position, time, validity, human performance

## Abstract

Advances in global navigation satellite system (GNSS) technology have resulted in smaller and more accurate GNSS receivers, which have become increasingly suitable for calculating instantaneous performance parameters during sports competitions, for example by providing the difference in time between athletes at any location along a course. This study investigated the accuracy of three commercially available GNSS receivers directed at the sports market and evaluated their applicability for time analysis in endurance racing sports. The receivers evaluated were a 1 Hz wrist-worn standalone receiver (Garmin Forerunner 920XT, Gar-920XT), a 10 Hz standalone receiver (Catapult Optimeye S5, Cat-S5), and a 10 Hz differential receiver (ZXY-Go). They were validated against a geodetic, multi-frequency receiver providing differential position solutions (accuracy < 5 cm). Six volunteers skied four laps on a 3.05 km track prepared for cross-country skiing, with all four GNSS receivers measuring simultaneously. Deviations in position (horizontal plane, vertical, direction of travel) and speed (horizontal plane and direction of travel) were calculated. In addition, the positions of all receivers were mapped onto a mapping trajectory along the ski track, and a time analysis of all 276 possible pairs of laps was performed. Specifically, the time difference between any two skiers for each integer meter along the track was calculated. ZXY-Go, CAT-S5, and GAR-920XT had horizontal plane position errors of 2.09, 1.04, and 5.29 m (third quartile, Q3), and vertical precision 2.71, 3.89, and 13.35 m (interquartile range, IQR), respectively. The precision in the horizontal plane speed was 0.038, 0.072, and 0.66 m s^-1^ (IQR) and the time analysis precision was 0.30, 0.13, and 0.68 s (IQR) for ZXY-Go, Cat-S5, and Gar-920XT, respectively. However, the error was inversely related to skiing speed, implying that for the low speeds typically attained during uphill skiing, substantially larger errors can occur. Specifically, at 2.0 m s^-1^ the Q3 was 0.96, 0.36, and 1.90 s for ZXY-Go, Cat-S5, and Gar-920XT, respectively. In summary, the differential (ZXY-Go) and 10 Hz standalone (Cat-S5) receivers performed substantially better than the wrist-worn receiver (Gar-920XT) in terms of horizontal position and horizontal speed calculations. However, all receivers produced sub-second accuracy in the time analysis, except at very low skiing speeds.

## Introduction

In most endurance sports such as cycling, running, rowing, or cross-country skiing, athletes move from a start point along a pre-defined track to finish in the shortest time possible. To provide athletes, coaches, and spectators with information describing the development of a race, intermediate times are commonly used to provide section time information. Such information provides some insight into the development of a race, but is limited, since changes in athletes’ performance often occur at a higher rate than the time elapsed in the individual sections. This limitation in analysis detail can be overcome if the athlete’s position is tracked instantaneously along the course from start to finish using wearable positioning devices such as global navigation satellite systems (GNSS) or local positioning systems (LPS). Instantaneous performance can be characterized by instantaneous time analysis, providing the relative difference in time between athletes at any location along the course. Such instantaneous time analysis allows the identification of events where athletes gain or lose time compared to their compatriots, and can even provide the rate at which time is gained and lost from start to finish of the entire race (Self et al., 2012; Bolger et al., 2015; Johnson et al., 2015; Gilgien et al., 2016; Losnegard et al., 2016; Sandbakk et al., 2016; Marsland et al., 2017). For cases where athletes follow a given track, differences in time between athletes are explained by differences in speed between the athletes. Hence, the measurement of instantaneous time and speed differences between athletes provides a more detailed performance analysis compared to the commonly used discrete intermediate time analysis. To allow instantaneous performance analysis, an athlete’s position and speed need to be tracked continuously during the race using methodologies that cause the least possible interference with the athlete’s sporting action, but that exhibit sufficient accuracy.

To track athletes’ positions and speed instantaneously, the primary technologies used are video-based tracking, LPS, and GNSS (Muthukrishnan, 2009). Video-based tracking is only applicable if the athletes are in the field of view of a camcorder throughout the race and are therefore not often used in racing and endurance sports. LPS is typically used for indoor sports but can also be used in outdoor sports that are held in limited space, such as on track loops (Self et al., 2012; Swarén et al., 2016; Swarén and Eriksson, 2017). GNSS does not have the two limitations described above and is therefore the most commonly applied wearable technology used to track athletes in outdoor sports.

The rapid development in GNSS technology over recent decades has substantially increased the number of different commercially available GNSSs suitable for sports applications. The GNSS receivers used in sports devices range from single-frequency chips incorporated in smartphones and wrist-worn training computers, to standalone units solely designed for athlete tracking and high-end geodetic receivers, which are typically carried on the athlete’s back and developed for purposes different from sports (tracking of planes, drones, etc.). Hence, the GNSS technologies applied in sports differ substantially in hardware and software quality and complexity (Supej and Cuk, 2014), which has an impact on measurement accuracy (Muthukrishnan, 2009). The major characteristics of GNSS properties that have impacts on position accuracy are: Antenna and GNSS board type; GNSSs used; GNSS frequencies used; and GNSS processing method (standalone, differential, precise point positioning, etc.) (Madry, 2015). Since GNSS receivers applied in sports should be small, light, and user-friendly, the manufacturers of wearable GNSS receivers need to find a trade-off between form factor, simplicity, system performance, and cost. Watches and smartphones obviously have limited space for a GNSS antenna and board and limited accuracy is expected, while receivers carried on the back can have a larger form factor. The number of GNSSs and satellites available has increased substantially over the last decade; with NAVSTAR GPS, GLONASS, Beidou, and the launching of Galileo, four functioning global systems are available. The number of GNSSs and satellites used also increases the accuracy and stability of position solutions for applications in sport (Gilgien et al., 2014b). Therefore, GNSS receivers used in sports increasingly tend to combine more than one GNSS. GNSS satellites send information on several frequencies. Use of multiple frequencies helps cancel out inaccuracies caused by the ionosphere. However, most GNSS receivers used in sports use only one frequency. Also, most GNSS receivers used in sports use only the GNSS information from the receiver carried by the athlete to calculate position (standalone solution). Combining the GNSS signal information from the receiver on the athlete with the GNSS information captured by a stationary GNSS receiver in close proximity (short baseline) substantially improves the position accuracy in dynamic applications (kinematic double difference method, hereafter called differential method) (Gilgien et al., 2014b). Further, position accuracy and robustness can be enhanced if GNSS data are combined with inertial measurement technology (IMU) (Skaloud and Limpach, 2003; Wägli, 2009; Fasel et al., 2016). GNSS solutions aimed at sports with reduced position accuracy requirements (i.e., most wrist-worn receivers or smartphones) apply single frequency analysis to one or two GNSSs in standalone mode (Terrier et al., 2000; Edwards et al., 2002; Townshend et al., 2008; Jennings et al., 2010a,b; Wisbey et al., 2010; Aughey, 2011; Clark et al., 2011; Macutkiewicz and Sunderland, 2011; Waldron et al., 2011; Bolger et al., 2015; Sandbakk et al., 2016). However, in sports with high demands for position accuracy, geodetic GNSS receivers are used in differential mode using multiple signal frequencies from one or several GNSSs to calculate position, speed, and acceleration (Larsson and Henriksson-Larsen, 2001; Skaloud and Limpach, 2003; Wägli, 2009; Andersson et al., 2010; Supej, 2010; Supej and Holmberg, 2011; Supej et al., 2012; Gilgien et al., 2013, 2014a,b, 2015a,b; Bucher Sandbakk et al., 2014; Nemec et al., 2014; Fasel et al., 2016; Kröll et al., 2016). Speed can be derived from time differentiation of the position data, or by using the Doppler principle on the GNSS signal (Zhang et al., 2006; Wang and Xu, 2011; Boffi et al., 2016), acceleration can be derived from position or measured with inertial sensors (Gilgien et al., 2014b; Supej and Cuk, 2014; Boffi et al., 2016). The accuracy of GNSS methods used in sports has been assessed for position (Townshend et al., 2008; Gilgien et al., 2014b, 2015b; Fasel et al., 2016), displacement (Townshend et al., 2008; Coutts and Duffield, 2010; Jennings et al., 2010a; Waldron et al., 2011; Hoppe et al., 2018), speed (Schutz and Herren, 2000; Witte and Wilson, 2004, 2005; Barbero-Alvarez et al., 2009; Coutts and Duffield, 2010; Waldron et al., 2011; Gilgien et al., 2015b; Boffi et al., 2016; Fasel et al., 2016), and acceleration (Gilgien et al., 2013, 2015b). However, most of these validations exhibited at least one of the following limitations: (1) Only one receiver was assessed per study, which does not allow a direct comparison between receivers/studies, since studies were conducted under different GNSS conditions and in different applications; (2) some studies applied a reference method that did not allow for instantaneous accuracy comparisons; (3) between-device reliability was not assessed. Further, only one of the validations focused on accuracy for split times and section times when validating a differential high-end receiver (Supej and Holmberg, 2011).

Therefore, the aim of this study was to assess three different classes of GNSS receivers that are frequently applied in sports for position, speed, and segment time accuracy in endurance racing sports. The receivers assessed were a 1 Hz low-grade wrist-worn receiver (Garmin Forerunner 920XT), a 10 Hz standalone receiver (Catapult Optimeye S5), and a 10 Hz differential GNSS receiver (ZXY Go). The accuracy of the three receivers was assessed by comparison with measurements using a high-end differential, multi-frequency, and multi-GNSS receiver (reference system) (Gilgien et al., 2013, 2014b, 2015b) for position, speed, and time analysis.

## Materials and Methods

### Participants and Test Protocol

The data presented in this study were collected during the Norwegian national cross-country skiing teams training camp at Sognefjell, Norway (61°33^′^53.79^′′^N, 7°59^′^51.54^′′^E, elevation 1434 m) on May 31, 2017. Six volunteers were recruited from the team’s support group. All participants were able skiers, but none of them were actively competing. The participants gave their written consent to participation, and the study was approved by the ethics board at the Norwegian School of Sport Sciences.

**FIGURE 1:**
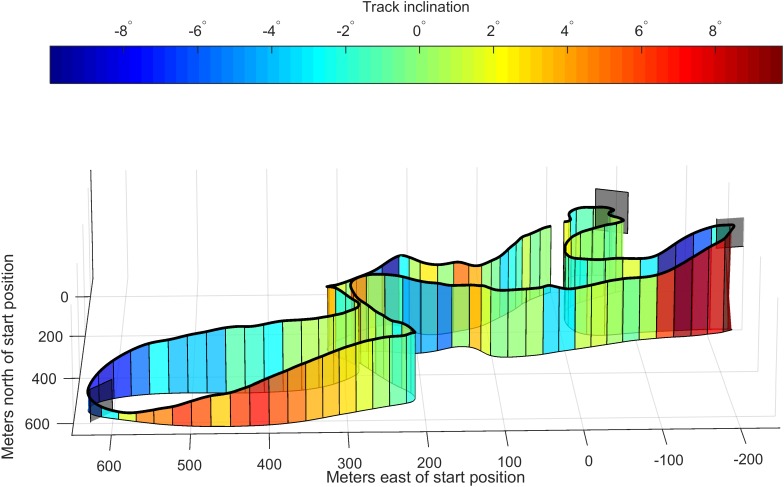
Track topography (vertical coordinates are multiplied by a factor of 3). The gray planes indicate the four sections to avoid local minima during the mapping procedure (see the section “Mapping trajectory”).

All participants were instructed to ski four laps of a specified track section (*L* = 3048 m, **Figure 1**). Between each lap, they were allowed a rest of approximately 1 min. They were instructed to ski at a pace close to their own typical racing speed. The participants were divided into two equally sized groups, with group 1 starting at approximately 10:15 a.m., and group 2 at approximately 4:45 p.m. Since GNSS conditions change with time (due to changes in constellations and atmospheric effects), the results were expected to vary between the two groups. The differences are highlighted in the results when these were substantial.

### Materials

**FIGURE 2:**
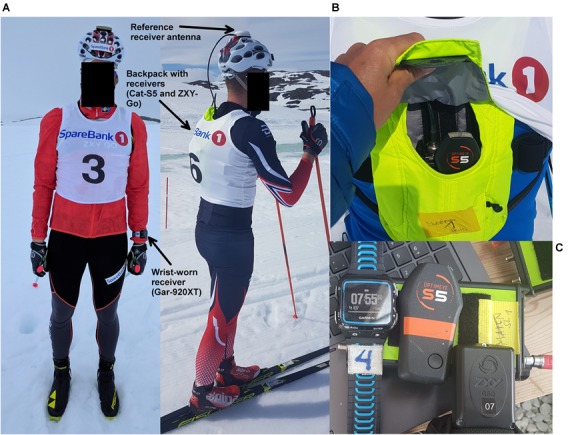
Experimental setup. **(A)** The reference system antenna was mounted on a bicycle helmet and coupled to the receiver in the backpack (hidden under the start bib). **(B)** Arrangement of receivers in the backpack. The ZXY-Go receiver was positioned just below the Cat-S5 receiver, and is not visible in this image. The Garmin receiver was worn on the wrist, and is also not visible. **(C)** The three evaluated receivers (on top, from left to right): Gar-920XT, Cat-S5, and ZXY-Go. Below: Reference receiver (Javad Alpha-G3T).

Each participant was equipped with one high-end differential GNSS receiver used as a reference, and the three GNSS receivers whose performance was to be evaluated. The reference system consisted of a differential multi-frequency and multi-GNSS receiver. Specifically, the base station consisted of a GNSS antenna (Grant-G3T, Javad, San Jose, CA, United States) and receiver (Alpha-G3T, Javad, San Jose, CA, United States) and was placed at the start of the ski track allowing for short baseline differential solutions. The athletes carried a GNSS antenna (G5Ant-2AT1, Antcom, Torrance, CA, United States, 160 g) mounted on a cycling helmet, and a GNSS receiver (Alpha-G3T, Javad, San Jose, CA, United States, 430 g) was carried in a small backpack (**Figure 2**). The sampling frequency was set to 10 Hz, which was the same frequency as the highest sampling frequencies of the evaluated receivers.

#### Evaluated Receivers

The Catapult Optimeye S5 (Firmware version 7.18, abbreviated as Cat-S5) has a 10 Hz GNSS with an external antenna, packaged with an IMU in a casing with dimensions: 96 × 52 × 13 mm. The sensor is intended to be worn in a harness on the torso and has a mass of approximately 67 g. In the current study it was placed in the athlete’s backpack, close to where it would be placed in the harness (**Figure 2**). The receiver was oriented in an erect position as recommended by the manufacturer.

A Garmin Forerunner 920XT (Garmin International, Inc., Olathe, KS, United States, abbreviated as Gar-920XT) was worn on the wrist. It samples at 1 Hz, has a mass of 61 g, and measures 45 × 55 × 13 mm.

**FIGURE 3:**
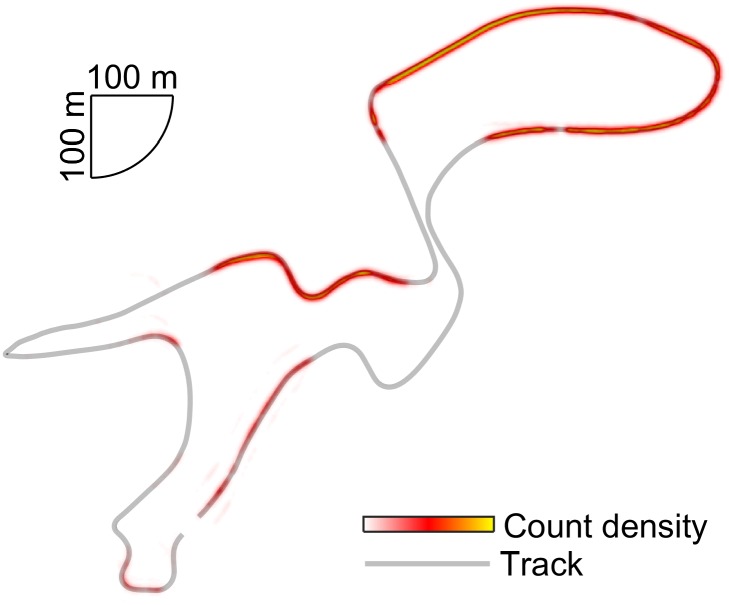
Heat map showing the spatial distribution of received ZXY-Go coordinates. The mapping trajectory is plotted in gray.

The ZXY-Go system (ChyronHego Norge A/S, Oslo, Norway) consists of tracking receivers intended to be worn in a harness on the torso. They measure 45 × 90 × 15 mm, have a mass of 63 g, and sample at 10 Hz. The current version of the receivers did not have local storage and data were sent in real time to a base station and were processed using a post-processing approach. This implies that position solutions were only calculated in periods when the receiver on the athlete was in the line of sight of the base station, which was not the case for the entire track (**Figure 3**). Future versions of this type of receivers tailored for the endurance sports market are expected to have local storage and/or a different radio transmission technology, avoiding the line-of-sight limitation. For post-processing, the GNSS data of the base station from the reference system were used. In the current study the receiver was placed in the athlete’s backpack, directly beside the Cat-S5 receiver, and was oriented based on the manufacturer’s recommendation. All three receivers apply single frequency (L1) analysis on GPS and GLONASS signals.

### Data Analysis

#### Reference System

Geodetic short baseline position solutions were calculated using dual frequency (L1 and L2) data from NAVSTAR GPS and the GLONASS satellite systems. The ambiguities of the differential position solutions were solved for all athletes and the entire time periods when athletes were skiing, using the kinematic algorithm of the geodetic post-processing software Justin (Javad, San Jose, CA, United States).

#### GNSS Position Solution Calculation

The conditions for GNSS measurements were excellent, with a position dilution of precision (PDOP) of 1.23 ± 0.15. Data from the ZXY-Go system were processed by ZXY staff according to their best practice principles, but were not filtered by the manufacturer. To reduce system bias, the GNSS base station data of the reference system were used, before they were sent to the authors as text files. GNSS solutions for Cat-S5 and Gar-920XT were calculated using their respective automated processing procedures and position results were exported to text files using Catapult Sprint software version 5.1.7, and Fit CSV Tool version 1.0.12.20, respectively. Data from the Cat-S5 and Gar-920XT were passed through their manufacturer’s proprietary filters. GNSS coordinates were expressed in the WGS84 coordinate frame. The Cat-S5 and Gar-920XT adjust for geoid height. The offset between orthometric height and GPS ellipsoidal height was calculated to be 46.022 m at the recording location, and was removed from the data (Wong and Gore, 1969). All subsequent analyses were conducted using Matlab R2017a (The MathWorks, Natick, MA, United States).

#### Time Synchronization

The ZXY-Go receivers were synchronized with the reference receiver using its GPS time stamps. Both Gar-920XT and Cat-S5 lacked support to export accurate GPS time. Therefore, they were first synchronized using their local time. In a second step, the synchronization offset (Δ*t*) from the reference receiver time was estimated from the slope of the position difference (Δ*s*) vs. speed (|*v*|) relationship:

Δs=|v|×Δt+k.

**FIGURE 4:**
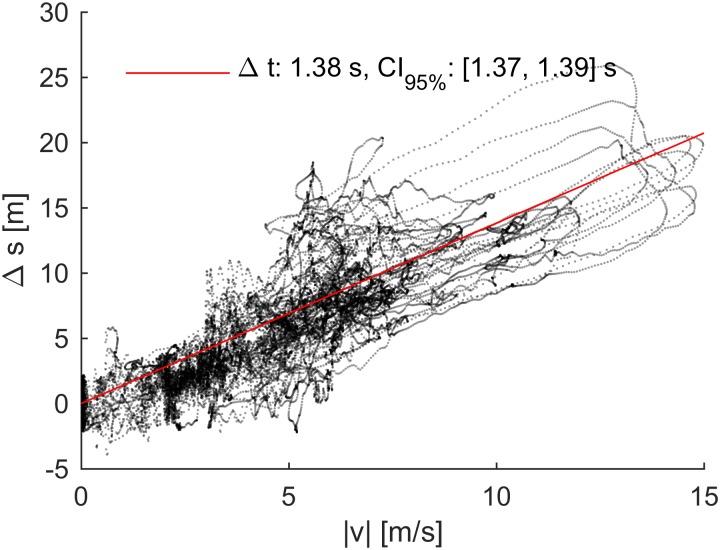
Example of the synchronization procedure used for the Gar-920XT and Cat-S5 receivers. If the receivers are not time synchronized with the reference receiver, it results in a position deviation in the direction of travel (Δ*s*) that increases linearly with skiing speed (|*v*|). The slope of the fitted line is an estimate of the synchronization offset.

Here Δ*s* refers to the position difference along the skiing direction, defined as the reference receiver’s horizontal plane velocity vector (**Figure 4**). The constant *k* was the systematic offset due to different antenna mounting positions (see the section “Correction of antenna mounting locations”). The regression was performed using a robust regression scheme with a bi-square weighting function and a tuning constant of 4.685.

#### Correction of Antenna Mounting Locations

The position data from each evaluated receiver were corrected for the typical offsets due to different anatomical mounting locations. Specifically, mean displacement vectors between markers positioned close to the different GNSS receivers’ positions were calculated based on optical motion capture marker positions from a previous study (Myklebust et al., 2015; Gløersen et al., 2017). A marker on the superior section of the head was used to represent the reference antenna location; a marker on the 10th thoracic vertebra was used to represent the two receivers in the backpack; and a marker located on the distal end of the left radius was used to represent the wrist-worn receiver. These vectors were added to the evaluated receiver position measurements (by transforming them to the East-North-Up coordinate frame). Specifically, the two receivers in the backpack were translated 33 cm forward (i.e., in the skiing direction), and 43 cm vertically upward. The wrist-worn device was translated 5 cm forward, 52 cm vertically upward, and 33 cm medially.

#### Mapping Trajectory

For the time analysis, the GNSS measurements were mapped onto a common trajectory (mapping trajectory). Because of the relatively narrow ski track (approximately 3 m), each athlete’s position in the direction perpendicular to the track was neglected. The trajectory computed from the reference system from the first lap of one of the subjects was used as a mapping trajectory. During this lap, our reference receiver had a fixed solution throughout the lap. The coordinates of the mapping trajectory were filtered with a 0.3 Hz low pass filter to remove frequencies caused by postural movements (see the section “Filtering and parameter calculation”). The filtered coordinates were then resampled to every integer meter and interpolated using a cubic spline.

The criterion for mapping onto the mapping trajectory was to minimize the Euclidean distance between a measured position and any given point along the mapping trajectory. Only the two horizontal coordinates were used for the mapping. To avoid situations where the mapping could suddenly jump to incorrect sections of the track (i.e., when two sections of the ski track passed close to each other), a piecewise mapping onto track segments of length max (10 m, Δ*t* × 20 m s^-1^) was performed. Here Δ*t* denotes the time since the last measurement. If there was a gap in the measurements of more than 5 s (relevant only for the ZXY-Go receivers), the mapping was done onto the whole mapping trajectory for the next position measurement. To minimize the likelihood of the solver finding only a local minimum, the track was partitioned into four sub-segments (**Figure 1**), and only the solution that returned the minimal Euclidean distance was kept.

The distance along the track was calculated from a piecewise linear curve through the mapping trajectory, starting at the first point and ending at the mapped position, with a node every integer meter. The start time was defined as the time of the reference system at the first sample after crossing the virtual start position, i.e., the first sample with a non-zero distance along the mapping trajectory.

#### Filtering and Parameter Calculation

The reference method measurements were filtered using smoothing splines weighted by their fixed/float status and predicted accuracy (Skaloud and Limpach, 2003) using a smoothing parameter of *p* = 0.995, as implemented in Matlab’s curve fitting toolbox. In a second filtering step, weights were set equal to zero for any samples having an acceleration norm greater than 25 m s^-2^, before reevaluating the smoothing spline. The smoothing spline was evaluated at the same times as the evaluated receivers, enabling an estimate of the reference receiver position at the time of each receiver’s position measurement.

Because the receivers were not positioned on the same anatomical locations, the GNSS positions of all receivers (including the reference receiver) were low pass filtered using a second order Butterworth filter with a cutoff frequency of 0.3 Hz. This cutoff frequency was determined based on the frequency spectrum of similar anatomical locations during treadmill ski skating. Specifically, the displacements of the head, hand, and 10th thoracic vertebra were determined using marker positions sampled at 250 Hz [data from previous study (Myklebust et al., 2015; Gløersen et al., 2017)]. The frequency spectrums of these measurements indicated that most of the signal’s power was confined to frequencies greater than 0.5 Hz. Velocity was calculated from differentiation of the position data using a five-point finite difference algorithm (Gilat and Subramaniam, 2008), and was filtered with the same 0.3 Hz low pass filter as the position measurements.

Horizontal plane speed was defined as the vector magnitude of the easting and northing velocity vector components. Speed along the mapping trajectory was obtained from numerical differentiation of the distance moved along the track using the same five-point finite difference algorithm (Gilat and Subramaniam, 2008), and was filtered using the same filter as the horizontal plane speed. Distance covered, i.e., the length of the trajectory traveled by the athlete, was calculated as the cumulative sum of Euclidean distances between each horizontal-plane GNSS position measurement. Hence, the distance covered could be calculated for each position measurement from the receivers. Due to gaps in the ZXY-Go position measurements (periods when the receivers did not have radio contact with the base station), distance covered could not be evaluated for the ZXY-Go receivers.

Both Catapult and Garmin calculate their own measurements of speed and distance covered using proprietary algorithms. Because of the filtering procedure specified in the previous paragraph, and to ensure a fair comparison against the ZXY-Go measurements, we decided to perform identical speed and distance covered calculations based on the GPS positions for all evaluated receivers. Deviations between proprietary measurements of speed or distance covered and the calculations performed in this study are briefly discussed later.

#### Time Analysis

To evaluate the time difference between athletes at identical positions along the mapping trajectory, the timestamps from each receiver were linearly interpolated to every integer meter along the mapping trajectory. Using the evaluated time points, both a split time (i.e., time from the common start time to any given position along the track) and a segment time (i.e., time between two given positions along the track) analysis were conducted. In both analyses, all 276 possible pairs of laps were analyzed.

In the split time analysis, the time difference between each pair of laps was calculated for every integer meter (starting at 10 m), disregarding measurements where one or more receivers were not recording. The segment time analysis also compared all possible pairs of laps, and the track was divided into equal length segments between 20 and 180 m, in steps of 20 m. The first segment started at the start line, and the subsequent segments started every 20 m. The time taken to complete the segment in each possible pair of laps was then compared. Segments where the ZXY-Go receiver was missing data at the end points were omitted from the analysis. Time analysis precision and accuracy of each GNSS method were then judged from the difference to the reference receiver results.

#### Statistics

Position errors were quantified as horizontal plane deviations (vector magnitude), vertical deviation, and the difference in distance measured along the mapping trajectory. The error distributions were visualized as histograms displaying the count density in each bin, where the bin spacing was chosen according to the Freedman–Diaconis rule (Freedman and Diaconis, 1981). The area of the histogram columns was normalized to unity. For the speed we calculated the difference in horizontal plane speed, and the difference in speed along the mapping trajectory. Robust statistical measures were used as descriptive statistics of the distributions. Specifically, median error (Med) and interquartile range (IQR) were used to quantify accuracy and precision, respectively. For the strictly positive horizontal plane deviations, distribution mode and third quartile (Q3) were used instead. In addition, the typical error of the estimate (TEE) was calculated as described by Hopkins et al. (2009) to allow comparison with studies where TEE was used. Measurements with more than three median absolute deviations from the median were considered outliers and were omitted from the calculation of TEE. The 95% confidence intervals for the statistics were calculated using a bootstrap approach valid for stationary time series (Politis and Romano, 1994). Each empirical distribution was subsampled block-wise using block lengths of *n*^2/3^, where *n* was the number of measurements in the empirical distribution. All statistics are presented in the text as 95% confidence intervals. Two of the laps contained short periods (a few seconds) where the reference receiver’s position ambiguities could not be resolved (i.e., the double difference ambiguities were float and accuracy not as good as when ambiguities are fixed). These two laps were omitted from the analyses of distances covered, because the reduced accuracy of the reference receiver during these time periods will affect measurements of distance covered throughout the lap.

## Results

Results are reported directly in the text or figures, but main results are summarized in **Tables 1**, **2**.

**Table 1:** Summary of the receiver errors observed in the current study.

		ZXY-Go	Cat-S5	Gar-920XT
δ*xy*	Mode (m)	0.53 [0.46, 1.21]	0.37 [0.34, 0.51]	2.72 [2.54, 3.28]
	Q3 (m)	2.09 [1.79, 2.55]	1.04 [0.95, 1.11]	5.29 [4.97, 5.54]
δ*z*	Med (m)	1.35 [-1.50, 2.61]	5.18 [4.70, 5.47]	-1.27 [-4.41, -0.54]
	IQR (m)	2.71 [2.26, 4.54]	3.89 [3.40, 3.59]	13.35 [10.80, 13.12]
	TEE (m)	2.57 [2.14, 2.62]	2.00 [1.76, 1.88]	5.11 [4.20, 5.03]
δ*l*	Med (m)	0.13 [-0.38, 0.39]	0.00 [-0.08, 0.04]	-0.32 [-0.59, -0.09]
	IQR (m)	0.95 [0.80, 1.51]	0.72 [0.65, 0.81]	4.31 [3.93, 4.66]
	TEE (m)	0.71 [0.60, 0.78]	0.51 [0.47, 0.54]	2.96 [2.81, 3.07]
δ|*v*|*_xy_*	Med (m s^-1^)	0.000 [-0.001, 0.002]	0.011 [0.010, 0.013]	0.087 [0.076, 0.097]
	IQR (m s^-1^)	0.038 [0.036, 0.043]	0.072 [0.070, 0.075]	0.658 [0.614, 0.835]
	TEE (m s^-1^)	0.027 [0.026, 0.028]	0.050 [0.049, 0.051]	0.484 [0.456, 0.505]
δ|*v*|*_l_*	Med (m s^-1^)	0.001 [-0.003, 0.004]	0.003 [0.002, 0.005]	0.015 [0.002, 0.027]
	IQR (m s^-1^)	0.046 [0.041, 0.067]	0.081 [0.077, 0.087]	0.752 [0.701, 0.927]
	TEE (m s^-1^)	0.035 [0.032, 0.036]	0.058 [0.056, 0.060]	0.551 [0.526, 0.575]
δ*d_s_*⋅*t^-^*^1/2^	Med (m s^-1/2^)	N/A	0.000 [-0.021, 0.015]	0.192 [0.044, 0.387]
	IQR (m s^-1/2^)	N/A	0.094 [0.085, 0.104]	0.743 [0.640, 0.800]
	TEE (m s^-1/2^)	N/A	0.060 [0.054, 0.060]	0.518 [0.445, 0.525]
Δ*t*	Median (s)	-0.00 [-0.03, 0.06]	-0.02 [-0.03, -0.02]	0.05 [0.02, 0.091]
	IQR (s)	0.30 [0.27, 0.40]	0.13 [0.12, 0.14]	0.68 [0.64, 0.75]
	TEE (s)	0.22 [0.20, 0.23]	0.09 [0.09, 0.09]	0.53 [0.52, 0.54]

*All errors are reported as the observed value with 95% confidence intervals. Distribution mode and third quartile (Q3) are reported for vector magnitudes, otherwise median, IQR, and TEE are used. Nomenclature: δ*xy*, horizontal plane position error; δ*z*, vertical position error; δ*l*, error along mapping trajectory; δ|*v*|*_xy_*, horizontal plane speed error; δ|*v*|*_l_*, error in speed along mapping trajectory; δ*d_s_t^-^*^1/2^, stochastic error in distance covered normalized by the square root of time elapsed since the start of the lap; δ*t*, split time error*.

**Table 2:** Accuracy (median error) and precision (IQR and TEE) of the evaluated receivers’ position measurements, calculated for each individual lap.

	Receiver	Median (m)	IQR (m)	TEE (m)	Outliers (%)
Easting	ZXY-Go	0.21 ± 1.25	0.36 ± 0.30	0.17 ± 0.09	19.3 ± 10.5
	Cat-S5	0.26 ± 0.45	0.43 ± 0.08	0.31 ± 0.06	0.8 ± 1.9
	Gar-920XT	2.09 ± 1.12	3.22 ± 0.53	2.40 ± 0.46	1.6 ± 1.4
Northing	ZXY-Go	-0.35 ± 1.15	0.61 ± 0.82	0.29 ± 0.33	19.3 ± 10.5
	Cat-S5	-0.25 ± 0.32	0.60 ± 0.16	0.40 ± 0.12	0.8 ± 1.9
	Gar-920XT	-0.43 ± 1.04	3.66 ± 0.67	2.54 ± 0.45	1.6 ± 1.4
Vertical	ZXY-Go	0.87 ± 4.47	1.16 ± 1.16	0.52 ± 0.55	19.3 ± 10.5
	Cat-S5	4.71 ± 2.00	0.92 ± 0.28	0.58 ± 0.15	0.8 ± 1.9
	Gar-920XT	-4.45 ± 7.02	1.57 ± 0.31	0.99 ± 0.16	1.6 ± 1.4

*The results are presented as the mean ± SD of all laps. When calculating TEE, measurements with a Euclidean difference exceeding three median absolute deviations from the median were considered outliers and were omitted from the analysis. The fraction of discarded measurements is presented in the last column*.

### Position Errors

**FIGURE 5:**
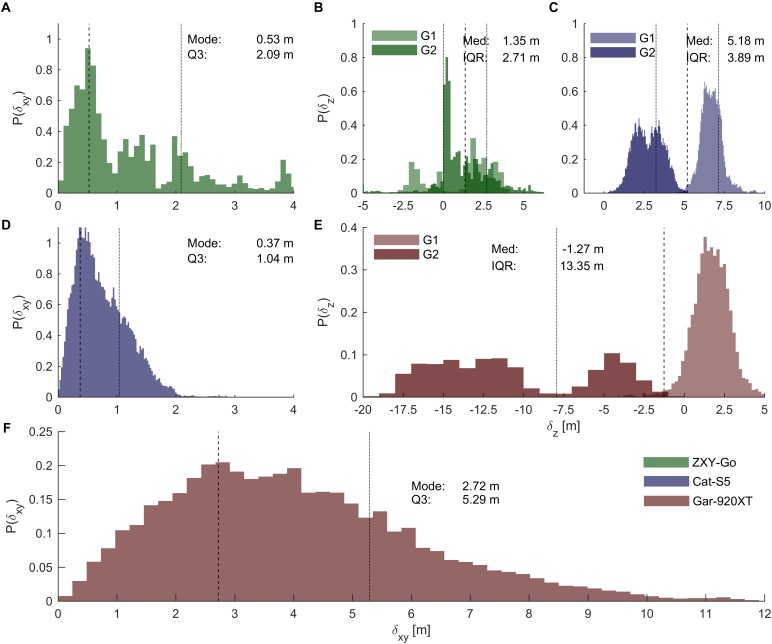
Position errors. **(A,D,F)** Distributions of horizontal plane errors for ZXY-Go, Cat-S5, and Gar-920XT, respectively. Dashed lines, distribution mode; dotted lines, third quartile. Horizontal axes are equally scaled. **(B,C,E)** Distributions of vertical error for ZXY-Go, Cat-S5, and Gar-920XT, respectively. Dashed lines, median error; dotted lines, IQR. Horizontal axes are equally scaled. The vertical error distributions of the two standalone receivers were clearly multi-modal, which suggests that the offset changed with time (as indicated by the different color saturation for the two groups of skiers, G1 and G2). Therefore, the analysis was also done on a lap-by-lap basis to evaluate accuracy and precision over shorter (∼9 min) time intervals (**Table 2**).

Typical horizontal plane position errors were similar for the ZXY-Go and Cat-S5 receivers (distribution modes [0.46, 1.21] and [0.34, 0.51] m, respectively), but the ZXY-Go exhibited a heavier tail (Q3 [1.79, 2.55] m compared to Cat-S5 [0.95, 1.11] m. See also **Figures 5A,D**). The Gar-920XT receiver showed substantially larger errors compared to the two other receivers (distribution mode [2.54, 3.28] m, **Figure 5F**).

The vertical position accuracy was best for ZXY-Go (distribution median [-1.50, 2.61] m), while Gar-920XT underestimated (median [-4.41, -0.54] m) and Cat-S5 overestimated (median [4.70, 5.47] m) vertical position slightly. However, as is apparent from **Figure 5**, the vertical accuracy changed substantially between the two groups of participants who started at different time points, especially for the Gar-920XT and Cat-S5 receivers. This implies that the IQR calculated from the aggregated data probably overestimates the expected variation over a typical race duration. Therefore, the median and IQR of the position deviations (both vertical and horizontal plane) were also calculated on each individual lap. The results of this analysis are presented in **Table 2**, and show that the IQRs of vertical deviation evaluated over a single lap were 1.16 ± 1.16, 0.92 ± 0.28, and 1.57 ± 0.31 m (mean ± SD of all laps) for ZXY-Go, Cat-S5, and Gar-920XT, respectively.

### Mapping Onto Mapping Trajectory

**FIGURE 6:**
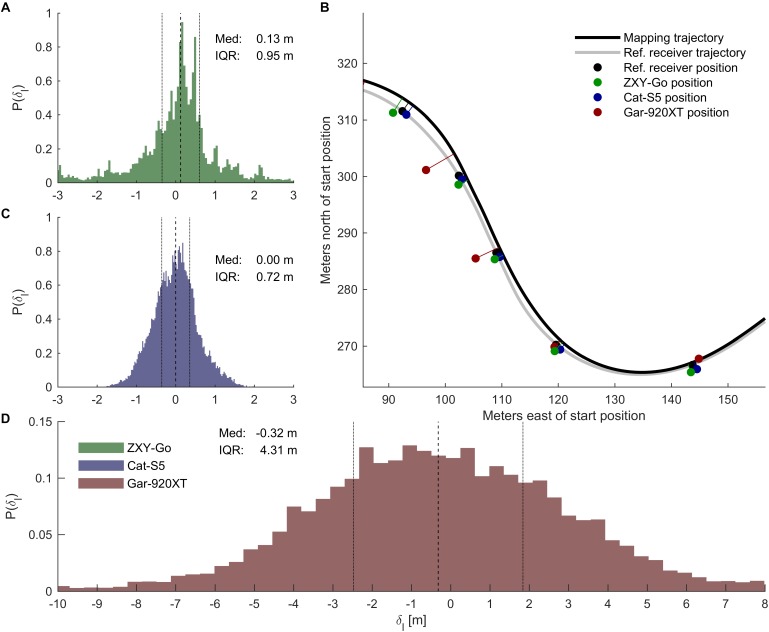
Mapping on mapping trajectory. **(B)** Section of the track showing how a subset of receiver coordinates were mapped onto the mapping trajectory (black line). The gray line shows the trajectory of the reference receiver for the given trial. The dots are the receivers’ coordinates sampled at the same time, with 3-s intervals (see legend for color specification). **(A,C,D)** Distributions of the mapped position errors, quantified as the distance to the reference receiver position along the mapping trajectory [see legend in (D) for color specification].

To reduce the position error, position data were mapped onto the mapping trajectory. The error in mapped position, measured as the distance between the receiver position and the reference position along the mapping trajectory, was similar for ZXY-Go and Car-S5 (IQR [0.80, 1.51] and [0.65, 0.81] m, respectively, **Figure 6**), while Gar-920XT exhibited a substantially larger error (IQR [3.93, 4.66] m). Example measurements and their corresponding mapped coordinates are plotted in **Figure 6B**.

### Speed Errors

**FIGURE 7:**
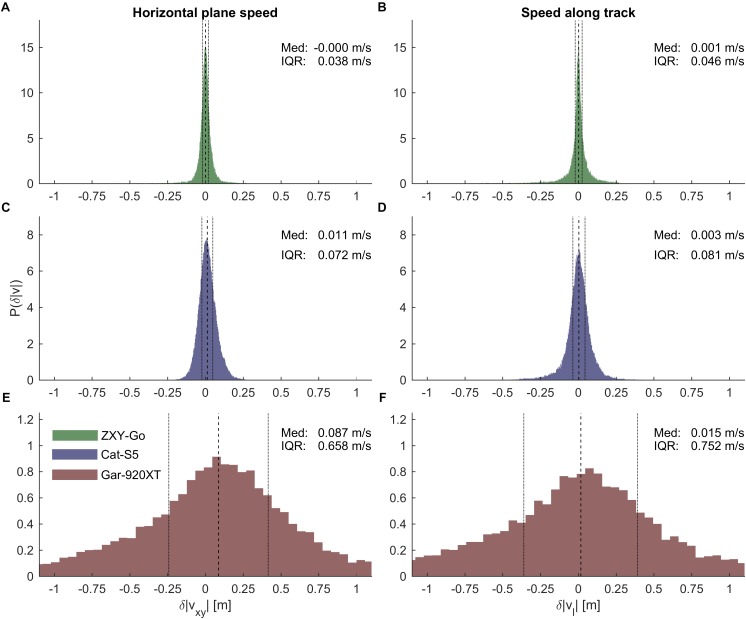
**(A,C,E)** Error distribution of horizontal plane speeds for ZXY-Go, Cat-S5, and Gar-920XT, respectively. **(B,D,F)** Error distributions of speed along the mapping trajectory for ZXY-Go, Cat-S5, and Gar-920XT, respectively. Dashed lines, median error; dotted lines, IQR. Horizontal axes are equally scaled. The ZXY-Go receiver showed the highest precision. Cat-S5 was comparable to the ZXY-Go receiver, while Gar-920XT showed substantially lower precision.

The horizontal plane speed error distributions are plotted in **Figures 7A,C,E**. The ZXY-Go receivers were most precise (IQR [0.036, 0.043] m s^-1^), followed by the Cat-S5 receivers (IQR [0.070, 0.075] m s^-1^) and Gar-920XT ([0.614, 0.835] m s^-1^). Both ZXY-Go and Cat-S5 were accurate (median [-0.001, 0.002] and [0.010, 0.013] m s^-1^, respectively), while Gar-920XT overestimated horizontal plane speed (median [0.076, 0.097] m s^-1^). Speed along the mapping trajectory (**Figures 7B,D,F**) showed similar precision to the horizontal plane distributions (IQR [0.041, 0.067], [0.077, 0.087], and [0.701, 0.927] m s^-1^ for ZXY-GO, Cat-S5, and Gar-920XT, respectively), but Gar-920XT accuracy was improved (median [0.002, 0.027] m s^-1^).

### Errors in Distance Covered

**FIGURE 8:**
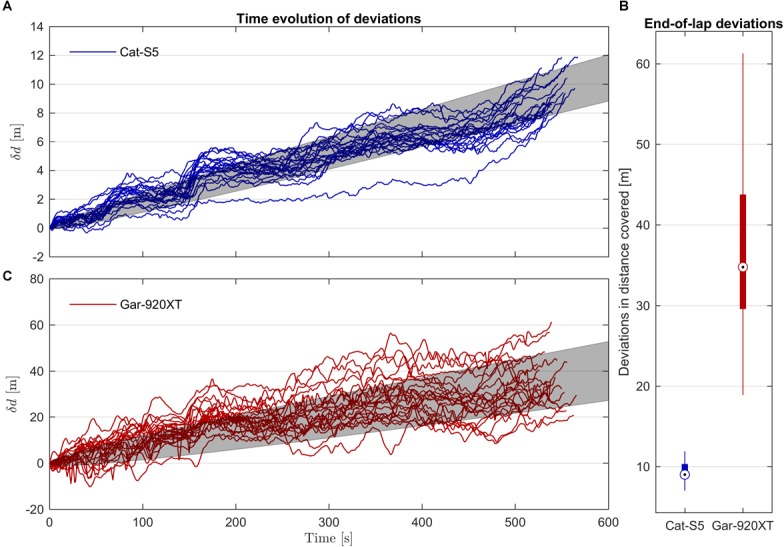
**(A, C)** Time evolution of the error in distance covered for Cat-S5 and Gar-920XT when compared to the distance covered by the reference receiver. Note the different scaling of the vertical axes. The errors show a linear drift term in agreement with the systematic difference in speed measurements between the evaluated receivers and the reference receiver. In addition, stochastic errors cause a deviation from the linear drift line which was approximately proportional to the square root of time. The gray shaded regions indicate the RMSD from the linear drift. **(B)** Box plot of the errors in distance covered evaluated at the end of each lap. Both Cat-S5 and Gar-920XT overestimated distance covered compared to the reference receiver, but Cat-S5 was substantially more precise.

Both Cat-S5 and Gar-920XT overestimated the distance covered during one lap compared to the length of the reference receiver trajectory (median errors 9.0 and 34.8 m, respectively). Precision was also better for Cat-S5 compared to the Gar-920XT (IQR 1.8 and 14.2 m, respectively), as apparent from **Figure 8B**. The variation (IQR) in distance covered between single laps (measured with the reference receiver) was 10.1 m. Hence, the precision of Cat-S5 is better than the differences that can be expected due to different trajectories used by the athletes over a 3.05 km course.

**FIGURE 9:**
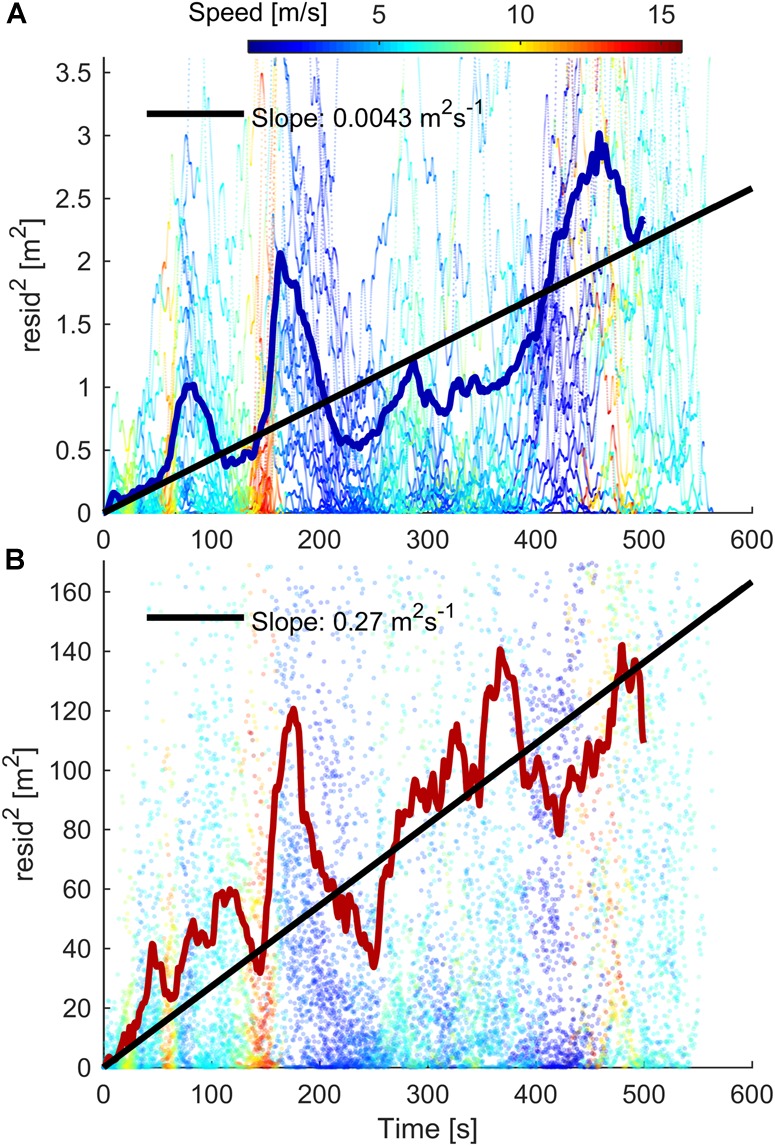
Scatter plots of the squared deviations from the linear drift line in **Figure 8** for Cat-S5 **(A)** and Gar-920XT **(B)**. The residuals are color-coded based on skiing speed. Solid colored lines show the mean-squared deviation at the given time. Black lines are the least squares fit (with zero *y*-intercept) to all the measurements. If the errors in speed were independent, identically distributed, and zero-mean, the expectation value of the squared error in distance covered would increase linearly with time (by Donsker’s theorem). In this experiment, these assumptions are violated due to changing receiver conditions and low-pass filtering of the trajectories. Nonetheless, the after subtraction of the linear drift, the error increases approximately linearly with time.

For both Cat-S5 and Gar-920, the time evolution of the error in distance covered was a combination of a linear drift which was equal to the mean error in speed, and a stochastic error (**Figures 8A,C**). The linear drifts were 1.7 and 67 mm s^-1^ for Cat-S5 and Gar-920XT, respectively. If the stochastic errors are independent, identically distributed, and zero mean, Donsker’s theorem implies that the mean-squared deviations from the linear trend line caused by systematic errors in speed should increase linearly with time. Although the assumptions of independence (due to the low pass filtering) and identical distributions (due to changing receiver conditions) are violated in this study, the mean-squared residuals still appeared to increase approximately linearly with time (**Figure 9**), except for some regions of the track. The color-coding in **Figure 9** suggests that changes in skiing speed could explain at least some of these deviations. The slope of the linear regression line of squared residuals was 0.0043 and 0.27 m^2^ s^-1^ for Cat-S5 and Gar-920XT, respectively (**Figure 9**). These findings imply that the expectation value for the error in distance covered increased linearly with time, and that the root mean-squared (RMS) deviation from the expectation value increased by the square root of time (**Figures 8A,C**). Therefore, the stochastic error in distance traveled divided by the square root of time elapsed was approximately constant throughout the lap. For the Cat-S5 and Gar-920XT receivers, the IQR of the stochastic error divided by the square root of time elapsed was [0.085, 0.104] and [0.64, 0.80] m s^-1/2^, respectively (**Table 1**).

### Split Time Analysis

**FIGURE 10:**
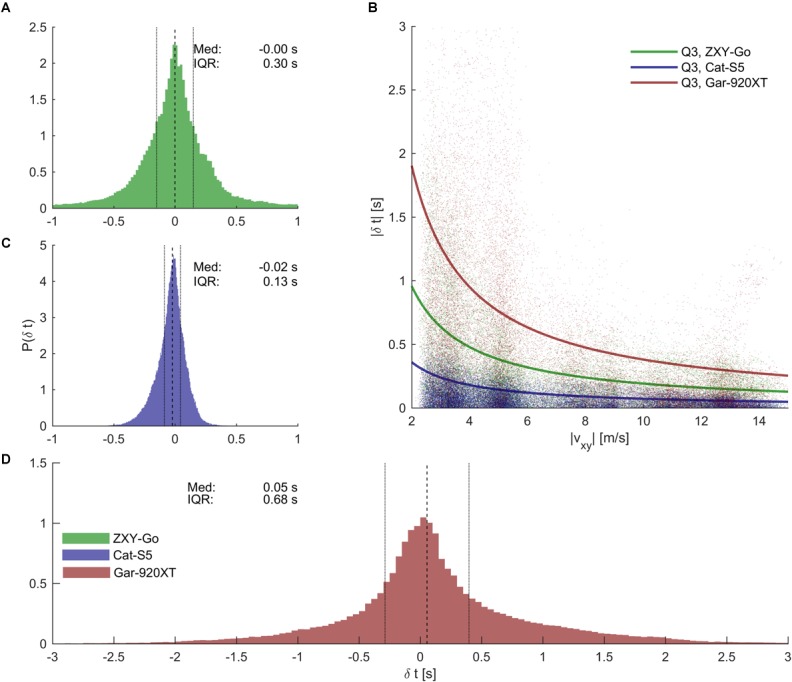
Time analysis errors. **(A, C, D)** Error distributions for time analysis errors for the three evaluated receivers [see legend in (D) for color specifications]. Dashed lines indicate median time error, and dotted lines IQR. Cat-S5 provided the most precise split times, followed by ZXY-Go and Gar-920XT. **(B)** Scatter plot showing that split time precision appeared to be inversely related to skiing speed (|*v_xy_*|) at the split time position. The solid lines are the hyperbolic functions | δ*t*| = *c*/|*v_xy_*| encompassing 75% of the samples (i.e., the third quartile).

The split time analysis resulted in precision (IQR) values of [0.27, 0.40], [0.12, 0.14], and [0.64, 0.75] s for ZXY-Go, Cat-S5, and Gar-920XT, respectively (**Figures 10A,C,D**). The split time error showed an inverse relationship with speed at the location where the split time was evaluated (**Figure 10B**).

### Segment Time Analysis

**FIGURE 11:**
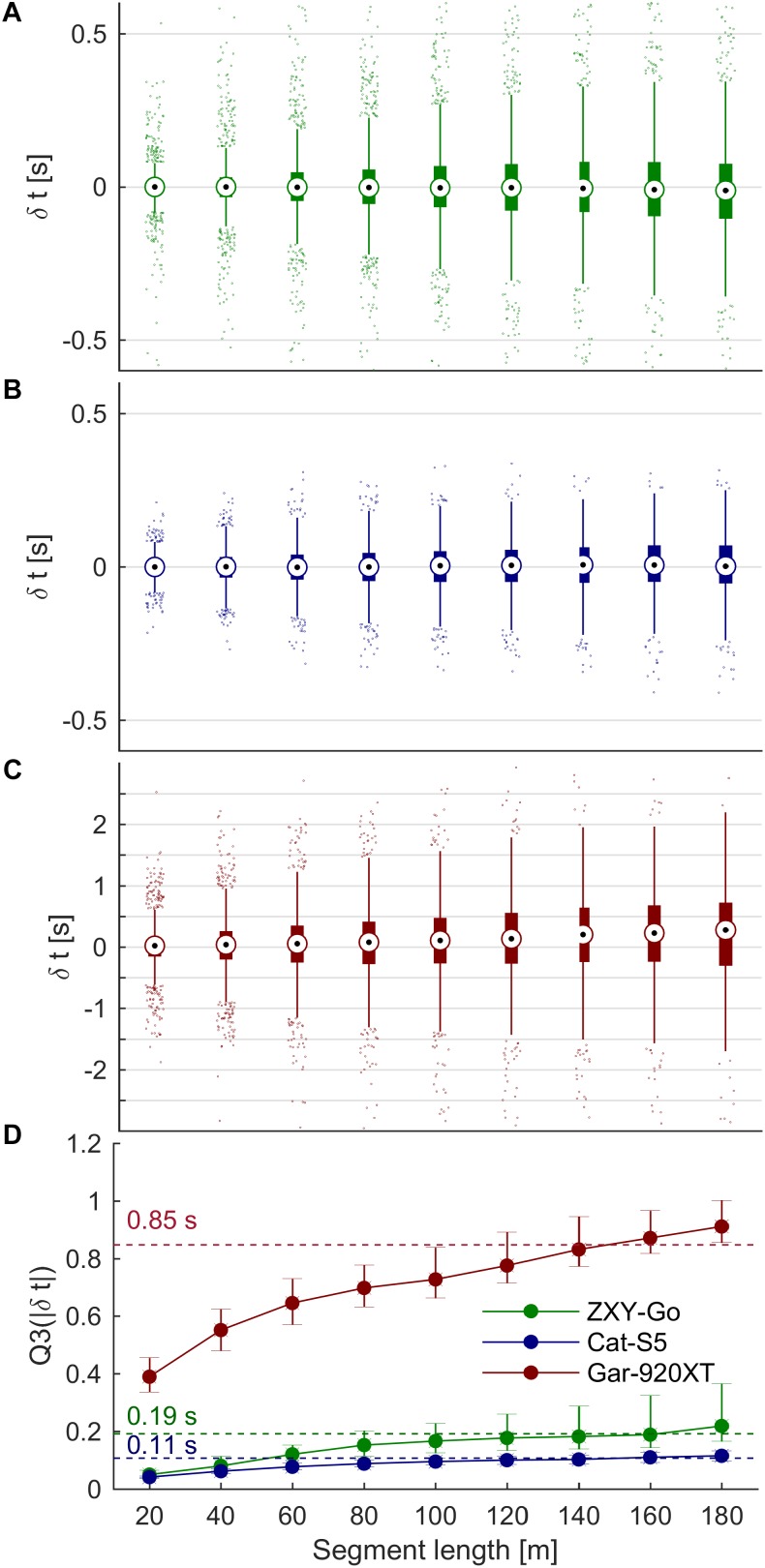
**(A–C)** Box plots of segment time error vs. segment length for ZXY-Go, Cat-S5, and Gar-920XT, respectively. Maximal whisker length is 1.5 × IQR. Horizontal grid lines are equally separated (0.5 s). **(D)** Third quartile of the absolute segment time error, with error bar indicating 95% CI. Segment time error increased with increasing segment length, but started to flatten out for segment lengths of 100 m, particularly for ZXY-Go and Gar-920XT. The dashed lines show the mean of segments with length >100 m.

Segment time error increased with segment length, but appeared to plateau for segment lengths >100 m, particularly for ZXY-Go and Cat-S5 (**Figures 11A–C**). When averaged over the four segment lengths >100 m, the ZXY-Go receiver’s absolute error (Q3) was 0.19 s, Cat-5S was 0.11 s, and Gar-920XT 0.85 s (**Figure 11D**).

### ZXY-Go Data Transmission

The ZXY-Go receivers successfully transmitted data on average for 33% (range: 21–44%) of the track length (**Figure 3**).

## Discussion

The aim of this study was to assess the accuracy of three different classes of GNSS receivers (1 Hz wrist worn, 10 Hz standalone, and 10 Hz differential), to measure position, speed, and segment time accuracy in endurance racing sports. The key findings of the study were: (1) there were substantial differences in accuracy between the three GNSS receivers, which need to be considered if applied to endurance racing sports; (2) split time error was strongly dependent on (and inversely related to) the athlete’s speed; and (3) segment time error increased with increasing segment length.

Few other studies have evaluated the performance of multiple GNSS receivers simultaneously in sports applications. One study evaluated three different receivers, but the experiment was aimed at typical team sports exercises (Coutts and Duffield, 2010). Furthermore, most sports-specific GNSS receiver validations have used straight line distances and optical speed traps (or chronometers) as reference measures for distance and average speed (Schutz and Herren, 2000; Townshend et al., 2008; Barbero-Alvarez et al., 2009; Coutts and Duffield, 2010; Waldron et al., 2011). The average speed determined from speed traps is not an ideal reference for evaluating GNSS receiver errors for three reasons: (1) during human locomotor tasks the GNSS receiver will seldom follow a straight line between two speed traps; (2) care must be taken to average over the same time interval, particularly if the sampling interval is not negligible compared to the averaging time; and (3) average speed provides only limited insight in sport applications. Therefore, to assess receiver position and speed the reference tracking system should be capable of measuring the true instantaneous trajectory of the receivers, using systems such as video-based tracking (Gilgien et al., 2013, 2014b, 2015b; Fasel et al., 2016), reflective marker-based tracking (Nedergaard et al., 2015) or, as in this study, a high-end GNSS receiver previously validated against video-based systems or similar. This study extends previous studies on sport-specific GNSS applications in three ways: (1) by comparing three different GNSS receiver technologies under the same conditions; (2) by comparing the trajectories in a dynamic situation where each receiver’s position could be validated instantaneously by comparison with the reference receiver’s smoothing spline; and (3) by investigating the accuracy of split times and segment times obtained from GNSS receivers aimed at the sports market, in a situation relevant for typical endurance racing sports (i.e., running, cycling, or cross-country skiing).

### Position Error

Position itself was not of primary interest in this study, as differences in choice of trajectory were not assessed in the performance analysis. However, position error was of interest since speed, split, and segment time are derived directly from position. Comparing the instantaneous position errors found in this study with the instantaneous position error found in a GNSS method validation in a racing sport application (Gilgien et al., 2014b), indicates that not only the GNSS method applied but also the receiver and antenna type and positioning of the GNSS antenna on the athlete play an important role in position error. The GNSS conditions (PDOP) were comparable between the studies, being very good in the alpine skiing study and excellent in the present study, while the dynamics were more pronounced in the alpine skiing study, resulting in overall more challenging measurement conditions in the Gilgien et al. (2014b) study. The present study agrees with the findings of Gilgien et al. (2014b) that position error can be reduced by using a differential solution (ZXY-Go) compared to a standalone solution (Gar-920XT) and shows that there are substantial differences between different standalone solutions. Although the Cat-S5 receiver and the ZXY-Go receiver were similar in many of the evaluated parameters, there was a clear indication that the ZXY-Go measurements were less robust than those obtained with the Cat-S5. This can be clearly seen from the heavier distribution tail in **Figure 5** and the number of outliers in **Table 2**. One explanation is that the ZXY-Go position solutions were not filtered by the manufacturer, leaving potential for further position accuracy enhancement (although all receivers were filtered using the same low pass filter in the data processing). Comparing the standalone GNSS solutions of the Gar-920XT and Cat-5S with the standalone GNSS code position method (E) in the Gilgien et al. (2014b) study, the position error was substantially larger for the Gar-920XT and smaller for Cat-S5. A more than 10 times larger error was found for the kinematic differential solution by ZXY-Go compared to a similar solution (Gilgien et al., 2014b) in the alpine skiing study. The fact that a geodetic high-end receiver was used in the alpine skiing study, combined with the large differences in position error for a given GNSS method between the present study and the Gilgien et al. (2014b) study, indicate that not only the GNSS method applied but also antenna and receiver size and quality are of importance for position accuracy in sport applications. Hence, the large position errors in the Gar-920XT might be associated not only with the heavily compromised antenna size and the receiver quality and processing procedure, but also with the mounting point on the athlete. The mounting point of the Gar-920XT, the wrist, which is swung forth and back continuously during skiing, causes changes in antenna orientation and GNSS signal reception, which challenges the GNSS processing (Weaver et al., 2015). GNSS signal shading by the athlete’s body may also reduce the performance of the Gar-920XT compared to the other receivers.

### Speed Error

With respect to horizontal plane speed, a study comparing five GNSS receivers ranging from a mobile phone receiver to a high-end differential receiver found larger errors in speed for a standalone wrist watch and a standalone handheld receiver than the present study could find (Supej and Cuk, 2014). The authors related the large error partly to the latency of about 2 s in the speed readings of these receivers. Latency effects were removed in the present study using the time synchronization procedure. The removal of latency could be an important reason for the reduced speed errors found in the present study compared to Supej and Cuk (2014). An alpine skiing study (Gilgien et al., 2015b), validating the speed of the center of mass approximation using GNSS and modeling, found larger speed errors than the present study. However, these were based on three-dimensional position data and included the error from the modeling approximation of the center of mass. A study conducted on a roller coaster, simulating the dynamics of racing sports, found errors in the range of cm/s for consumer-grade receivers targeted to dynamic applications (Boffi et al., 2016), which is similar to the results of the present study.

We found only minor differences in the precision of speed measurements between horizontal plane speed and speed along the mapping trajectory. However, speed accuracy was improved by the mapping procedure, particularly for Gar-920XT. The speed used in the current study was deduced by differentiating the GPS positions (before or after mapping onto the mapping trajectory). Most GNSS receiver manufacturers calculate speed using other (proprietary) algorithms. For the Gar-920XT, the manufacturer’s speed estimate was similar in precision to the speed reported in the current study, but was more accurate (exhibiting only a trivial overestimation). The Cat-S5 can calculate speed based on the Doppler principle. The precision was similar to the speed reported in the current study, but it tended to overestimate speed slightly compared to our reference receiver. A likely explanation for this overestimation is the low pass filter applied to the GNSS coordinates of the reference receiver in the current study. This filtering process removes high frequency movements within each technique cycle, effectively shortening the true trajectory of the receiver prior to differentiation. In contrast, the Doppler method measures speed directly based on the receiver’s true trajectory. Therefore, the two speed measurements are not directly comparable even when treated with the same low pass filter.

**FIGURE 12:**
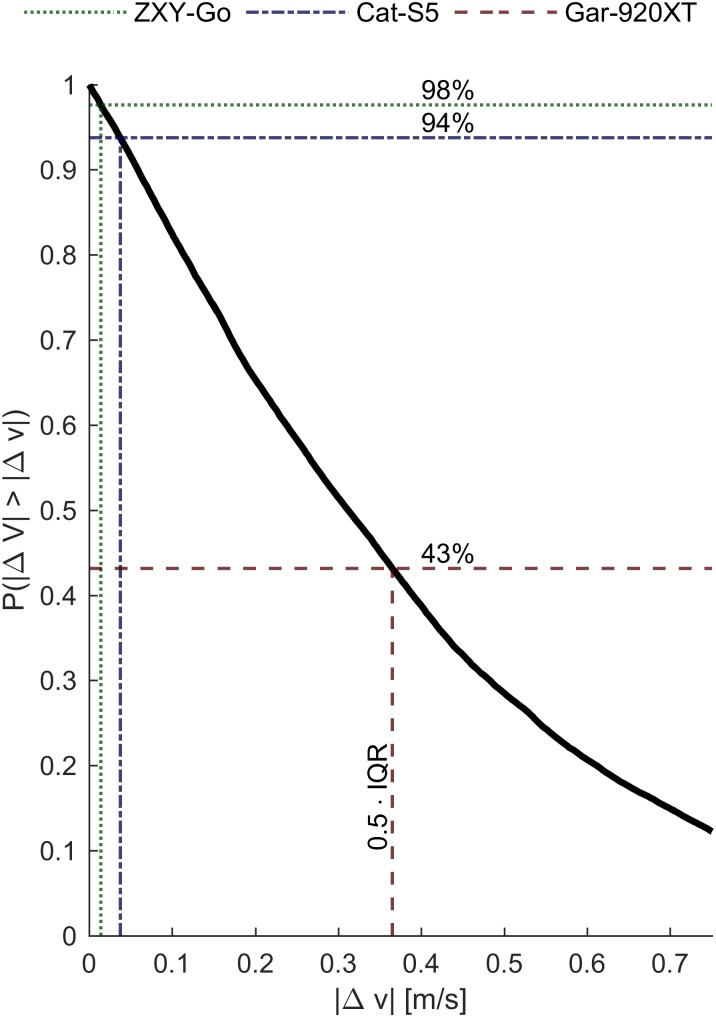
Kaplan–Meier curve showing the estimated probability of observing speed differences (Δ*V*) greater than Δ*v* on different laps, but at the same location and for the same participant. Half the IQR of the horizontal plane speed errors is indicated for the three evaluated receivers. It is clear that Gar-920XT cannot provide sufficiently precise estimates of speed to discriminate typical differences in speed within each athlete, while ZXY-Go and Cat-S5 can.

The accuracy requirements to assess instantaneous speed differences during a race would obviously depend on the specific sport. To elucidate these requirements for cross-country ski racing, we compared the intra-athlete variation in instantaneous speed on successive laps. Specifically, we compared the speed on laps 1 vs. 3, and 2 vs. 4, evaluated at every integer meter along the track (**Figure 12**). From these data it was clear that the Gar-920XT receiver would fail in most instances to report reliable instantaneous speed differences (speed differences were greater than 0.5 IQR for only 43% of the measurements). In contrast, both the ZXY-Go and the Cat-S5 could be used to differentiate typical speed differences observed in this study (speed differences greater than 0.5 IQR in 98 and 94% of the measurements, respectively).

### Error in Distance Covered

The findings of the current study suggest that errors in the distance covered exhibit a drift that is linear in time and equal to the errors in speed measurement, and a stochastic drift with an expectation value that increases with the square root of time. For many applications, the latter effect will be the most important, for instance when comparing several trials using the same GNSS receiver. The results also show that measurements of the distance covered by a GNSS receiver cannot be used for the time analysis purposes in the current study, because differences in the length of the athletes’ trajectories accumulate over time. This problem cannot be wholly resolved by using more accurate position measurements, but requires a common mapping trajectory, as used in the time analysis in the current study.

### Split and Segment Time Error

An obvious, but important, prerequisite for using GNSS for time analysis is that a “meaningful difference” in performance is encompassed by a position difference greater than the receiver error, for two athletes starting simultaneously. Therefore, and as the results of this study imply, it is beneficial to segment the track so that the athlete has a high speed when passing the segment boundaries. For instance, for the Gar-920XT receiver, split time accuracy (Q3) was 1.90 s where the speed was 2 m s^-1^, and 0.25 s where the speed was 15 m s^-1^ at the evaluated position.

Furthermore, the error in the time analysis decreases with decreasing segment length. This is most likely due to correlated position errors at both segment end points, resulting in a cancelation of the errors, given that the track is relatively straight. It is important to note that if the evaluated segment of the track includes a sharp turn and the track points in approximately opposite directions at the endpoints, the errors will most likely no longer cancel. However, as a minimal criterion, short track segments should be avoided for time analysis.

Time analysis accuracy requirements are typically a function of segment duration, since the relative time difference is almost independent of competition duration (Stöggl and Müller, 2008). However, for endurance racing sports, individual choices of pacing strategy (de Koning et al., 2011) or differences in technical skill level can result in considerable differences over relatively short segments. The results for section time accuracy presented in the current study may help to define sections for analysis in which the applied GNSS system provides the required accuracy.

### Methodological Considerations

#### Validity of the Reference System

Under circumstances with excellent conditions for GNSS measurements (PDOP < 2), the reference system used in this study has previously been shown to have a position accuracy of about 5 cm (Gilgien et al., 2014b). This is small, but not negligible, compared to the distribution modes in **Figure 5**. Furthermore, the four GNSS antennas were mounted on different anatomical locations. We corrected for the average position differences by translating the evaluated receiver’s position measurements, but individual differences in anthropometrics and changes in posture will introduce deviations from the ideal situations of identical antenna positions. The magnitude of these errors can be estimated by calculating the distances from the head-mounted antenna to the translated wrist or thoracic antennas. Using the measurements from a previous study (Myklebust et al., 2015; Gløersen et al., 2017), this error was estimated to be 0.26 m (RMS) for the wrist-worn receiver, and 0.09 m (RMS) for the backpack-mounted receivers. This is about 10 and 20% of the distribution modes (**Figure 5**) for the wrist-worn and backpack-worn receivers, respectively. It is therefore likely to have had some influence on the calculated errors. The error in speed derived from the reference receiver has not been validated directly, but was estimated to be <10 mm s^-1^ using numerical simulations based on the expected position uncertainties (5 cm) and the filtering procedure applied in the current study. This estimate is in agreement with the findings of Boffi et al. (2016), who evaluated speed using a lower-end receiver than the reference receiver used in the current study.

#### Mapping Procedure

The mapping of the measured positions onto a common trajectory was necessary for a successful time analysis, because the distance covered by the individual athletes during each lap varied from lap to lap. We chose to omit the vertical position dimension when performing the mapping procedure. Because the vertical dilution of precision (DOP) is often substantially higher than the horizontal DOP, including the vertical position is likely to reduce the mapped position accuracy.

This mapping procedure would also be useful in calculations of the mechanical work rate of the athletes. On a track with substantial inclines, the energy required to raise the center of gravity is often the dominant work athletes need to perform. Having accurate measurements of vertical position is a key prerequisite to making reliable estimates of this work. The validity of mechanical work rate estimations using GNSS receivers was not addressed in the current study, but it should be considered in future studies.

#### Limitations

Because the conditions for GNSS measurements during these experiments were excellent, our findings reflect a best-case situation. Therefore, further assessment in sub-optimal conditions (higher PDOP and more challenging signal multipath conditions) is necessary to investigate how the different receiver methods are affected by changes in measurement conditions. Furthermore, the accuracies reported here cannot be generalized to sports with substantially higher speeds or accelerations (e.g., motor sports or alpine skiing). Large vertical speed and displacement can also cause the receiver accuracy to deteriorate, because of changes in the atmospheric signal transmission properties.

The differential receiver (ZXY-Go) evaluated in the current study did not have local storage and, due to frequent lack of line of sight, lost the data transfer link between receiver on the athlete and the base station, leading to loss of data in those time periods. However, both these issues can be resolved in future receivers. Because small carrier-phase differential receivers have the potential to substantially increase the three-dimensional accuracy of position tracking in sports applications, we decided to include this receiver in the study even if the current version is not suitable for time analysis in cross-country skiing.

Between-device reliability and test–retest reliability were not addressed in the current study, but could be of interest for further research.

## Summary And Conclusion

The results of this study revealed substantial variation in the accuracy obtained using commercially available GNSS receivers aimed at sports applications, which should be considered when a GNSS receiver is chosen for a specific application in endurance racing sports. In summary, the ZXY-Go (differential) and Cat-S5 (standalone) receivers performed substantially better than the wrist-worn Gar-920XT receiver for horizontal plane position, speed, and time analysis calculations. The receiver’s horizontal plane speed errors suggested that the ZXY-Go and Cat-S5 can detect typical instantaneous speed differences in cross-country ski racing, while the Gar-920XT cannot.

## Ethics Statement

This study was carried out in accordance with the recommendations of the Norwegian Centre for Research Data. The protocol was approved by the Ethics Committee at the Norwegian School of Sport Sciences. All subjects gave written informed consent in accordance with the Declaration of Helsinki.

## Author Contributions

ØG, JK, and MG: conception and design, and data collection. ØG and MG: data analysis. MG: manuscript draft introduction. ØG: manuscript draft methods and results. ØG, MG, and JK: manuscript draft discussion. All authors contributed to manuscript revision, read, and approved the submitted version.

## Conflict of Interest Statement

The authors declare that the research was conducted in the absence of any commercial or financial relationships that could be construed as a potential conflict of interest.
